# Psychometric evaluation of novel hyperphagia questionnaires in a real-world setting for patients with a rare MC4R pathway disease

**DOI:** 10.1186/s13023-025-03948-1

**Published:** 2025-08-22

**Authors:** Jeremy Pomeroy, Usha G. Mallya, Min Yang, Caroline Huber, Alexandra Greatsinger, Ella Hagopian, Andrea M. Haqq

**Affiliations:** 1 Marshfield Clinic Research Institute, Marshfield, WI USA; 2 Rhythm Pharmaceuticals, Inc., Boston, MA USA; 3 Analysis Group, Inc., Boston, MA USA; 4 University of Texas, Austin, TX USA; 5 Division of Pediatric Endocrinology, University of Alberta, Edmonton, AB Canada

**Keywords:** Hyperphagia, MC4R pathway diseases, Bardet-Biedl syndrome, Obesity, Psychometric evaluation, Validity, Patient-reported outcome

## Abstract

**Background:**

There are no validated measures to assess hyperphagia associated with rare MC4R pathway diseases, such as Bardet-Biedl Syndrome (BBS). Symptoms of Hyperphagia© (SoH) and Impacts of Hyperphagia© (IoH) are novel questionnaires designed to assess signs and symptoms of hyperphagia and their impacts on patients and caregivers. We evaluated the psychometric performance of the caregiver-versions of the SoH: Caregiver (Observer-reported) and IoH: Caregiver (Observer-reported and Self-reported subscales).

**Results:**

Reliability and validity were evaluated using data from a multi-country cross-sectional survey of adult caregivers of patients with BBS experiencing hyperphagia and obesity. Other instruments included were Impact of Weight on Quality of Life (IWQOL)-Kids (Parent Proxy), PROMIS Scale Global Health of Caregiver, Revised Impact on Family Scale (RIOFS), and Work Productivity and Activity Impairment. 242 eligible caregivers completed the survey. Exploratory factor analysis identified 1 factor per subscale. Strong internal consistency was observed for IoH: Caregiver (Observer) (Cronbach’s a = 0.66) and IoH: Caregiver (Self) (a = 0.72) and moderate for SoH: Caregiver (Observer) (a = 0.40). Moderate-to-strong correlations were observed with school days missed and all domains of IWQOL-Kids except Physical Comfort (range = 0.315–0.573, *p’s <* 0.001). Known-groups indicated significantly worse SoH: Caregiver subscores for patients using appetite suppressants or implementing more weight management approaches (6–10 vs. ≤5 or > 10). Caregivers reporting greater strain on RIOFS items and worse mental health had worse IoH subscores.

**Conclusions:**

The SoH: Caregiver and IoH: Caregiver demonstrated preliminary validity, reliability, and consistency in a real-world setting. Research is underway to further validate these measures for use in clinical trials for BBS and other MC4R pathway-related diseases associated with obesity.

## Background

The melanocortin-4 receptor (MC4R) pathway, primarily located in the hypothalamus, is responsible for regulating hunger and energy expenditure, and consequently body weight. Impaired signaling in this pathway due to rare genetic variants or injury to the hypothalamic region can lead to hyperphagia and severe obesity [1]. Rare MC4R pathway diseases of obesity include proopiomelanocortin, proprotein convertase subtilisin/kexin type 1, or leptin receptor deficiency, or Bardet-Biedl syndrome (BBS). In particular, BBS is a syndromic MC4R pathway disease with hallmark symptoms of hyperphagia and early-onset severe obesity [2]. Affected patients often experience a constellation of symptoms and/or complications including retinal dystrophy, postaxial polydactyly, obesity, genital anomalies, renal anomalies, and learning disabilities [2–4]. Hyperphagia, i.e., pathological insatiable hunger with accompanying food-seeking behaviors, is one of the early and persistent characteristic symptoms of BBS and can lead to adverse emotions (e.g., frustration, sadness, guilt) and strained family dynamics for patients and their caregivers due to the characteristics and consequences of hyperphagia, including the patient’s inability to control their desire to eat, shortened duration of satiety, preoccupation with food, and other associated abnormal food-seeking behaviors [5]. Early onset of hyperphagia and obesity also increases the risks of developing comorbidities and cardiometabolic abnormalities such as hyperlipidemia, type 2 diabetes mellitus, hypothyroidism and polycystic ovarian syndrome [6]. 

Timely identification and effective management of hyperphagia can have a profound, sustained positive impact on the lives of the patients affected by such disorders and their caregivers. A few patient-reported outcome (PRO) instruments have been developed and validated for assessing hyperphagia in patients with Prader-Willi Syndrome (e.g., Dykens’ Hyperphagia Questionnaire, Hyperphagia Questionnaire for Clinical Trials) and in the general population (e.g., Night Eating Diagnostic Questionnaire) [7–10]. These instruments assess the severity of food-related preoccupations and problems, as well as condition-specific correlates such as lack of emotional regulation in Prader-Willi Syndrome and poor sleep quality in Night Eating Syndrome. With their respective intended focuses, none of these instruments was developed for or has been validated in patients with disrupted MC4R pathways. Therefore, such instruments do not adequately reflect signs, symptoms, and impacts of hyperphagia associated with patients suffering from MC4R pathway-related disorders, or enable clinicians to accurately distinguish between hyperphagia and normal hunger. Importantly, given the early onset of hyperphagia in patients with conditions like BBS, caregivers’ observations play a crucial role in recognizing the signs and communicating to medical professionals.

In order to informatively assess the impact of hyperphagia due to rare MC4R pathway diseases on patients and caregivers, four novel questionnaires were developed to assess signs and symptoms of hyperphagia and impacts of hyperphagia on patients and caregivers. These questionnaires include: (1) Symptoms of Hyperphagia: Caregiver Version (observer-reported), (2) Impacts of Hyperphagia: Caregiver Version (observer-reported and self-reported), (3) Symptoms of Hyperphagia: Patient Version (self-reported), and (4) Impacts of Hyperphagia: Patient Version (self-reported) (all © 2021, Rhythm Pharmaceuticals, Inc. All Rights Reserved). The development of the questionnaires was informed by the literature and semi-structured qualitative interviews with clinicians with expertise in managing hyperphagia in patients with BBS [11], as well as with patients and their caregivers [12]. In this study, we evaluated psychometric performance of the caregiver versions of Symptoms of Hyperphagia and Impacts of Hyperphagia in caregivers of patients with BBS.

## Methods

### Data source

Data came from the CAREgiver Burden in BBS (CARE-BBS) study, a cross-sectional multi-country survey study of adult caregivers of patients with BBS who live with obesity and hyperphagia. Details on survey design, eligibility criteria, and recruitment process of CARE-BBS have been previously reported [13–15]. Caregivers were recruited from Canada, Germany, United Kingdom (UK), and United States (US) who had cared for a patient with BBS for ≥ 6 months and were able to read and understand the local language of their country. To be eligible, the patients in their care needed to have a diagnosis of BBS, caregiver-observed hyperphagia, and a history of obesity (i.e., current presence of obesity or ever having a weight in the ≥ 95th percentile for the patient’s age and sex). Professional caregivers (i.e., those paid for their time to care for the patient with BBS) and caregivers of patients who were enrolled in a clinical trial at the time of the survey or during the preceding 6 months were excluded from the study. Eligibility criteria were confirmed based on caregiver’s report via a secure web-based survey portal.

In addition to responses on the two hyperphagia caregiver version questionnaires, the CARE-BBS study also collected data using other measures including the Impact of Weight on Quality of Life–Kids (IWQOL-Kids) (Parent Proxy), the Revised Impact on Family Scale (RIOFS), Work Productivity and Activity Impairment (WPAI): BBS-Caregiver, and PROMIS Scale–Global Health of Caregiver. The survey also collected caregiver-reported data on patient characteristics, including clinical characteristics, medication and weight-management strategy use, educational attainment and behaviors, as well as caregivers’ socio-demographics and health-related quality of life [14, 15]. 

### Survey measures

#### Symptoms of hyperphagia: caregiver version (Observer-reported)

The Symptoms of Hyperphagia (SoH): Caregiver Version [SoH: Caregiver (Observer)] consists of 5 items to measure the frequency of hunger-related patient behaviors observed by the caregiver in a 24-hour recall period. The five items are “try to negotiate or argue for more food than provided”, “eat extremely quickly”, “sneak or take food without permission”, “wake up asking or looking for more food during the night”, and “ask for more food after just finishing a meal or snack”. The response options include never (= 0), one to two times (= 1), and three times or more (= 2). SoH: Caregiver (Observer) is scored as the total score divided by the number of items answered, ranging from 0 to 2. Higher scores indicate greater hyperphagia severity (Table 1).

**Table 1:** Characteristics of Symptoms of Hyperphagia and Impacts of Hyperphagia – Caregiver version

	Symptoms of Hyperphagia: Caregiver Version (Observer-Reported)	Impacts of Hyperphagia: Caregiver Version (Observer-Reported)	Impacts of Hyperphagia: Caregiver Version (Self-reported)
**Items**	How often did the person in your care… 1. Try to negotiate or argue for more food than provided 2. Eat extremely quickly 3. Sneak or take food without permission 4. Wake up asking or looking for more food during the night 5. Ask for more food after just finishing a meal or snack	To what extent did the person in your care’s hunger negatively affect his/her… 1. Sleep 2. Mood/emotion 3. School 4. Leisure or recreational activities 5. Relationships with family/friends	To what extent did the person in your care’s hunger negatively affect your… 1. Sleep 2. Mood/emotion 3. Work 4. Leisure or recreational activities 5. Relationships with family/friends
**Recall period**	Past 24 h	Past 7 days	Past 7 days
**Response options**	0 = Never 1 = One to two times 2 = Three times or more	0 = Not at all 1 = A little 2 = Moderately 3 = A great deal	0 = Not at all 1 = A little 2 = Moderately 3 = A great deal
**Scoring** ^**a**^	Total score divided by the number of items answered; range: 0–2	Total score divided by the number of items answered; range: 0–3	Total score divided by the number of items answered; range: 0–3

^**a**^Higher scores indicate greater severity and impact

#### Impacts of hyperphagia: caregiver version

The Impacts of Hyperphagia (IoH): Caregiver Version consists of two subscales, observer-reported [IoH: Caregiver (Observer)] and self-reported [IoH: Caregiver (Self)], each with five items measuring the extent to which hyperphagia affects the patient’s or caregiver’s respective sleep, mood or emotions, school, leisure or recreational activities, and relationships with family or friends over the past 7 days. The response options are not at all (0), a little (1), moderately (2), and a great deal (3). The subscales are scored separately for patient impact and caregiver impact, as the total score divided by the number of items answered, ranging from 0 to 3. Higher scores indicate greater impact of hyperphagia on daily activities and emotions (Table 1).

#### Impact of weight on quality of life (IWQOL)-Kids© (Parent-Proxy)

The IWQOL-Kids (Parent Proxy) [16, 17] is a 27-item parent proxy questionnaire and includes four domains (“Physical Comfort”, “Body Esteem”, “Social Life” and “Family Relations”) to measure the impact of weight on kids’ health-related quality of life. The instrument uses a 5-point Likert scale (1 “always true” to 5 “never true”). Item responses across all four domain scores are summed and scaled to generate a total score ranging from 0 to 100. Higher scores represent better health-related quality of life.

#### Revised impact on family scale (RIOFS)

The RIOFS [18] has 15 items that assess a family member’s perception of the effect of a child’s chronic condition on family life. The instrument has been shown to have strong face validity and favorable psychometric evaluations, including construct validity [19]. The RIOFS generates a total score ranging 0–60 using a 4-point Likert scale (“Strongly Agree”, “Agree”, “Disagree”, “Strongly Disagree”) along with two subscales capturing Personal Strain (score range 0–24) and Familial/Social Impact (score range 0–36). Higher scores indicate that the patient’s chronic condition has a greater impact on family life.

#### PROMIS scale v1.2–Global Health - Adult

The PROMIS Scale v1.2–Global Health [20] has 10 items assessing an adult’s overall health. This instrument generates a Global Mental Health score and a Global Physical Health score. All items except one use 5-point Likert scales (5 “Excellent” to 1 “Poor”; 5 “Completely” to 1 “Not at all”; 5 “Never” to 1 “Always”, and 5 “None” to 1 “Very severe”). One item that measures pain is on an 11-point numeric rating scale whereby 0 represents “No pain” and 10 represents “Worst pain imaginable.” This instrument was used to assess caregivers’ general health. A T-score was calculated using response pattern scoring; a higher T-score represents better overall health.

#### Work productivity and activity impairment (WPAI): BBS-Caregiver

The WPAI: BBS-Caregiver is an adapted questionnaire [21] to measure the impact of caregiving on work productivity (e.g., hours missed from work and actual hours worked) and regular daily activities due to caregiving for someone with BBS. The WPAI has 6 items and a recall period of “the past 7 days.” The WPAI produces 4 domain scores: absenteeism, presenteeism, total work impairment, and total activity impairment, ranging from 0 to 100% whereby a higher percentage indicates greater work or activity impairment.

### Statistical analysis

Internal consistency reliability was assessed by evaluating Cronbach’s alpha for the SoH and the IoH subscales, respectively. Cronbach’s alpha coefficient > 0.6 was considered to be acceptable for the initial exploratory evaluation [21]. Item-to-total correlation was further assessed for reliability using Pearson’s product-moment correlation coefficient. A correlation coefficient value between 0.3 and 0.7 was considered to be a preferred range to ensure that items are sufficiently correlated with reasonable heterogeneity across the items [22]. Floor and ceiling effects were examined through assessing the item response distribution for the scales; clustering at the minimum or maximum end of each scale would indicate the presence of floor or ceiling effects, respectively.

Exploratory factor analysis (EFA) was used to test the factorial structure of the SoH: Caregiver and IoH: Caregiver questionnaires using maximum likelihood as the extraction method. The Kaiser-Meyer-Olkin (KMO) and Bartlett’s test of significance were performed to assess sampling adequacy. Values that were considered to be acceptable to move forward to examine EFA were KMO ≥ 0.5 and Bartlett’s test of significance < 0.05.[23] An eigenvalue above 1 was used as the criterion for retaining a tool-specific factor with an extraction value above 0.4 [23]. Convergence of the questionnaires was assessed with existing validated measures such as PROMIS Scale–Global Health of Caregiver, IWQOL-Kids (Parent Proxy), and WPAI: BBS-Caregiver.

Lastly, known-groups validity was assessed by comparing score differences between groups defined based on the clinical relevance and supported by existing literature. Levene’s tests were conducted to assess the equality of variances across the groups. If the homogeneity of variance across the subgroups was rejected (*p* < 0.05), a Mann–Whitney U test was used to compare the mean scores between the subgroups. Otherwise, two-sample t-test was used to compare mean and standard deviation between the groups. Cohen’s d was calculated by d = (Mean_1_ - Mean_2_)/SD_pooled_. Cohen’s guidelines for interpreting effect sizes—small (0.1 to < 0.3), moderate (0.3 to < 0.5), and large (≥ 0.5) —were applied, with a large effect size (*r* ≥ 0.5) considered indicative of convergent validity [24]. 

## Results

### Caregiver and patient characteristics

A total of 242 caregivers from Canada (*n* = 62), Germany (*n* = 61), the United Kingdom (*n* = 59), and the United States (*n* = 60) met the eligibility criteria and completed the survey. Caregiver characteristics were comparable across countries and analyses were conducted using the entire pooled sample. The mean (SD) age of the caregivers was 41.9 (6.7) years, with 54% being male and 86% being married or in a domestic partnership. A large majority of the caregivers were either the father (51.7%) or mother (41.7%) of the patient with BBS. The mean (SD) age of patients with BBS was 12.0 (3.7) years, and patients were predominately (64%) male, with a mean (SD) modified BMI Z-score of 4.1 (4.5). The mean (SD) patient age at which the caregivers first noticed symptoms of uncontrollable hunger was 8.2 (3.6) years old, and 95% of caregivers reported that hyperphagia contributed to the diagnosis of BBS in these patients. Caregivers reported using an average of 8 weight management approaches for patients with BBS under their care: 162 (66.9%) used between 6 and 10 approaches, 46 (19.0%) used 11 or more approaches and the remaining 34 (14.0%) used 5 or fewer approaches [14, 15]. 

#### Reliability

Internal consistency reliability tests revealed Cronbach’s alpha coefficients of 0.40, 0.66, and 0.72 for the SoH: Caregiver (Observer), IoH: Caregiver (Observer), and IoH: Caregiver (Self), respectively, indicating the two IoH subscales had acceptable internal consistency reliability for the initial exploratory evaluation (Table 2). With Cronbach’s α < 0.5, the SoH: Caregiver (Observer) showed a lack of sufficient interrelatedness among the items. On the other hand, all of the item-total Pearson’s correlation coefficients were within the range of 0.30 and 0.70, except for three items in the IoH: Caregiver (Self) where the item-total correlations were greater than 0.70, suggesting some redundancy in the concepts within the IoH: Caregiver (Self) scale (Table 2). No floor or ceiling effects were detected for any of the scales (Tables 3 and 4).

**Table 2:** Internal consistency reliability and item-to-total correlation reliability

	Cronbach’s Alpha	Pearson’s Correlation
**Symptoms of Hyperphagia: Caregiver (Observer)**	0.40	
Try to negotiate or argue for more food than provided		0.510*
Eat extremely quickly		0.514*
Sneak or take food without permission		0.521*
Wake up asking or looking for food during the night		0.555*
Ask for more food after just finishing a meal or snack		0.610*
**Impacts of Hyperphagia: Caregiver (Observer)**	0.66	
Sleep		0.647*
Mood or emotions		0.636*
School		0.603*
Leisure or recreational activities		0.671*
Relationships with family or friends		0.685*
**Impacts of Hyperphagia: Caregiver (Self)**	0.72	
Sleep		0.666*
Mood or emotions		0.630*
Work		0.717*
Leisure or recreational activities		0.711*
Relationships with family or friends		0.719*

**Table 3:** Item response distributions for floor and ceiling effects – Symptoms of Hyperphagia

	Mean (SD)	Never	1–2 Times	3 + Times
**Symptoms of Hyperphagia: Caregiver (Observer)**	1.2 (0.4)			
Try to negotiate or argue for more food than provided		10.3	60.7	28.9
Eat extremely quickly		18.6	40.1	41.3
Sneak or take food without permission		19.4	48.8	31.8
Wake up asking or looking for food during the night		11.6	59.5	28.9
Ask for more food after just finishing a meal or snack		14.5	55.4	30.2

**Table 4:** Item response distributions for floor and ceiling effects – Impacts of Hyperphagia

	Mean (SD)	Not at all (%)	A little (%)	Moderately (%)	A great deal (%)
**Impacts of Hyperphagia: Caregiver (observer)**	1.6 (0.6)				
Sleep		7.0	39.3	39.7	14.0
Mood or emotions		14.5	29.8	35.1	20.7
School		10.7	32.6	40.9	15.7
Leisure or recreational activities		12.8	25.6	42.1	19.4
Relationships with family or friends		12.4	36.8	37.2	13.6
**Impacts of Hyperphagia: Caregiver (Self)**	1.6 (0.6)				
Sleep		9.5	33.9	38.8	17.8
Mood or emotions		11.6	31.8	40.1	16.5
School		13.6	33.1	38.0	15.3
Leisure or recreational activities		14.0	33.1	33.5	19.4
Relationships with family or friends		16.5	35.5	34.3	13.6

#### Construct validity

KMO and Bartlett’s tests confirmed sample adequacy for conducting EFA where the KMO and Bartlett’s test values were 0.53 for IoH: Caregiver (Observer) (*p* < 0.001), 0.71 for IoH: Caregiver (Self) (*p* < 0.001), and 0.75 for SoH: Caregiver (Observer) (*p* < 0.01). EFAs confirmed one factor structure for all scales. Eigenvalues for the extracted single factor were 2.03 for the IoH: Caregiver (Observer), 2.81 for the IoH: Caregiver (Self), and 0.82 for the SoH: Caregiver (Observer). The single factor extracted for the SoH: Caregiver (Observer) had an eigenvalue less than 1.0, suggesting current items within this scale may not be efficient in capturing the one underlying concept in this setting.

The convergence assessment revealed appropriate directionality of the questionnaires (i.e., higher/worse scores were correlated with worse outcomes captured by other relevant instruments/domains), which further supports construct validity (Table 5). The highest correlations were observed between the total scores of the IoH subscales and the IWQOL-Kids’ (Parent Proxy) domain, Family Relationships (both *r* > 0.5), indicating that worse impacts of hyperphagia on patients and caregivers as reported by the caregivers also had worse scores captured in the Family Relationships domain. The majority of the other measures had moderate correlations with the IoH subscales. For example, caregivers reporting greater impacts of hyperphagia on patients and on themselves tended to also report a greater impairment in their work productivity and daily activities as measured by the WPAI. For the SoH: Caregiver (Observer) questionnaire, the highest correlation was observed with the number of school days missed in the past 7 days (*r* = 0.438), indicating that patients with worse hyperphagia symptoms tended to miss more days from school. Moderate correlations were also observed with body esteem, family relationships, social life as captured through IWQOL-Kids (Parent Proxy). Moderate negative correlations were observed for the age of the person with BBS across all three sub-scales, indicating lower impact scores with increasing age.

**Table 5:** Convergence validity via Spearman’s rank-order correlations between SoH/IoH questionnaires and other measures

Instrument	Domain	SoH: Caregiver (Observer)	IoH: Caregiver (Observer)	IoH: Caregiver (Self)
**IWQOL-Kids (Parent Proxy)** ^**§**^	Total Score	-0.363***	-0.492***	-0.447***
	Body Esteem	-0.346***	-0.443***	-0.345***
	Family Relationships	-0.345***	-0.516***	-0.573***
	Physical Comfort	-0.278***	-0.381***	-0.297***
	Social Life	-0.315***	-0.416***	-0.359***
**RIOFS**	Total Impact		0.351***	0.370***
	Personal Strain		0.380***	0.385***
	Familial/Social Impact		0.274***	0.295***
**WPAI: BBS Caregiver**	Total Productivity Impairment		0.448***	0.435***
	Total Activity Impairment		0.417***	0.425***
**Days of school missed in past 7 days**		0.438***	0.454***	0.381***
**Age of person with BBS**		-0.168**	-0.206**	-0.205**
**Age of caregiver**		-0.058	-0.206**	-0.169**

BBS, Bardet-Biedl Syndrome; IoH, Impacts of Hyperphagia; IWQOL-Kids, Impact of Weight on Quality of Life; QoL, Quality of life; RIOFS, Revised Impact on Family Scale; SoH, Symptoms of Hyperphagia; WPAI, Work Productivity and Activity Impairment

**P < 0.01; ***P < 0.001

*Notes*: For the IWQOL-Kids (Parent Proxy), a higher score indicates a higher (i.e., better) quality of life and/or less burden. For all other instruments, a higher score indicates a lower (i.e., worse) quality of life and/or more burden

#### Known-groups validity

To evaluate known-groups validity, anchor variables were identified based on their conceptual relevance to hyperphagia. For the SoH: Caregiver (Observer), significant differences in mean total scores were observed across adjacent categories of weight management approaches (≤ 5, 6–10, ≥ 11), with mean scores of 0.98, 1.17, and 1.31, respectively (∆ = 0.19 and 0.14; both *p* < 0.05; Table 6). Further, caregivers who reported current use of appetite suppressants for patients (e.g., bupropion/naltrexone, phentermine/topiramate, liraglutide, semaglutide) reported significantly higher hyperphagia symptom scores for their patients (*p* < 0.001). Caregivers who agreed with the RIOFS statements “Nobody understands the burden I carry” and “It is hard to find a reliable person to take care of my child” reported a higher hyperphagia symptom burden for the patients under their care (both *p* < 0.03). The Cohen’s d effect sizes showed moderate to large effects, with the largest effect found when caregivers agreed to the RIOFS statement of “Nobody understands the burden I carry” (*r* = 0.86).

**Table 6:** Known-groups validity comparisons – Symptoms of Hyperphagia: Caregiver (Observer)

	Symptoms of Hyperphagia: Caregiver (Observer)		
	Group Difference (95% CI)	*P*-Value	Cohen’s D
**# of weight management approaches used**			
*≤ 5 vs. 6 ≤ x ≤ 10*	0.19 (0.06, 0.33)	0.006**	0.55
*6 ≤ x ≤ 10 vs. ≥ 11*	0.14 (0.02, 0.25)	0.024*	0.42
**Current use of appetite suppressant** (*Yes vs. No*)	0.20 (0.11, 0.28)	< 0.001***	0.78
**RIOFS: “Nobody understands the burden I carry”** (*Yes vs. No*)	0.21 (0.11, 0.30)	< 0.001***	0.86
**RIOFS: “It is hard to find a reliable person to take care of my child”** (*Yes vs. No*)	0.10 (0.00, 0.20)	0.028*	0.38

RIOFS, Revised Impact on Family Scale

*P < 0.05, **P < 0.01, ***P < 0.001

For the IoH subscales, caregivers who experienced more stressors in life and caregivers of patients with behavioral challenges reported worse hyperphagia impact scores for patients and for themselves (Table 7). Agreement with RIOFS items such as “Nobody understands the burden I carry”, “Sometimes I feel like we live on a rollercoaster”, and “It is hard to find a reliable person to take care of my child” was consistently associated with significantly greater reported impacts due to hyperphagia (all *p* < 0.05). Similarly, caregivers who reported fair or poor mental health as measured by PROMIS Global Health also experienced a greater impact of hyperphagia as measured by the IoH-Caregiver (self) (*p* < 0.05). Additionally, caregivers of patients who woke up more during the night due to uncontrollable hunger and caregivers of patients who had been told that they have a behavioral or conduct problem or a developmental delay also reported significantly greater hyperphagia impacts on patients and on themselves than caregivers who did not report these behavioral challenges or symptoms of hyperphagia. All Cohen’s d effect sizes showed moderate to large effects, with the largest effect found for the IoH: Caregiver (Self) and IoH: Caregiver (Observer) when caregivers reported that their BBS patients currently use an appetite suppressant (Cohen’s d = 1.53 and 1.14), respectively.

**Table 7:** Known-group validity comparisons – Impacts of Hyperphagia: Caregiver (Observer and Self)

	Impacts of Hyperphagia: Caregiver (Observer)			Impacts of Hyperphagia: Caregiver (Self)		
	Group Difference (95% CI)	*P*-Value	Cohen’s D	Group Difference (95% CI)	*P*-Value	Cohen’s D
**Number of times wake up in the night due to uncontrollable hunger in past 7 days** (< 5 vs. ≥ 5)	0.17 (0.02, 0.33)	0.061	0.41	0.18 (0.01, 0.35)	0.040*	0.40
**Current use of appetite suppressant** (*Yes vs. No*)	0.45 (0.32, 0.59)	< 0.001***	1.14	0.61 (0.47, 0.75)	< 0.001***	1.53
**RIOFS: “Nobody understands the burden I carry”** (*Yes vs. No*)	0.38 (0.23, 0.53)	< 0.001***	0.89	0.24 (0.08, 0.41)	0.002**	0.51
**RIOFS: “Sometimes I feel like we live on a roller coaster”** (*Yes vs. No*)	0.21 (0.05, 0.37)	0.012*	0.49	0.26 (0.09, 0.43)	0.004**	0.56
**RIOFS: “It is hard to find a reliable person to take care of my child”** (*Yes vs. No*)	0.23 (0.07, 0.38)	0.003**	0.54	0.27 (0.10, 0.44)	0.002**	0.58
**PROMIS Global Health of Caregiver: Mental Health** (*Fair/poor vs. excellent/very good/good*)	0.25 (0.05, 0.45)	0.011*	0.63	0.23 (0.01, 0.45)	0.039*	0.52

RIOFS, Revised Impact on Family Scale

*P < 0.05, **P < 0.01, ***P < 0.001

## Discussion

As an early-onset syndromic MC4R pathway disease, BBS places substantial burden on patients and their families, and negatively affects their wellbeing [25]. Beyond the demand of carrying out daily activities and coordinating medical care visits, caregivers often face substantial psychological distress and family strain due to social stigma and guilt [26]. As hyperphagia can result in multifaceted consequences on the lives of patients and caregivers [14, 15], timely recognition of the signs, symptoms and impacts is crucial for clinical management of these patients’ medical condition. However, the lacking effective tools to differentiate between hyperphagia and typical sensations of hunger is a gap in educating clinicians of the need for timely recognition, diagnosis, and ultimately effective management hyperphagia. Providing observer-reported and self-reported tools specifically to assess hyperphagia in rare MC4R pathway diseases could help close such gaps and facilitate productive communication between caregivers and medical professionals to improve patient outcomes.

Prior research on the symptom burden and impacts of hyperphagia and obesity in BBS has largely been qualitative. Much of the work has articulated the social stigma that caregivers experience when caring for a patient living with obesity, and found that caregivers often experienced negative social judgment or blame due to their association with a person who is overweight or obese [27–29]. Parents of children with BBS reported experiencing negative social perceptions and feeling blamed, devalued and judged by others for their child’s obesity, resulting in recurrent emotions of anger, frustration, and helplessness among the parents [26]. Lack of sufficient awareness of BBS among healthcare providers, as well as logistical difficulties that come with coordinating caregiving activities, can also lead to feelings of distress and frustration in these caregivers [30]. 

To date, there have been no validated measures to systematically quantify the burden of hyperphagia in this population. The SoH and IoH are novel questionnaires developed precisely for this purpose, with concepts elicited through interviews with patients and caregivers. These questionnaires are brief and straightforward and can be completed and scored easily at home for self-monitoring or during a clinical visit. The results can inform and facilitate a meaningful patient/caregiver–clinician dialogue about symptoms and disease control to improve clinical management of the condition.

Results of our psychometric evaluation support reliability and validity of the IoH: Caregiver Version, with one factor extracted for each of the subscales (for self-reported impacts on themselves and for observed-reported impacts on the persons with BBS). Internal consistency reliability of the two subscales were supported with Cronbach’s α > 0.5. The correlations with the IWQOL-Kids’ (Parent Proxy) domain Family Relationships and WPAI domains showed the reasonable directional associations and hence supported convergence validity. Findings from the known-groups validity analysis further confirmed the validity of the IoH: Caregiver Version where caregivers indicated worse impacts of hyperphagia on the patients and on the caregivers they also experienced greater distress in their lives.

On the other hand, the evaluation of the SoH: Caregiver Version showed less compelling evidence for the reliability, given a smaller than 1.0 eigenvalue from the exploratory factor analysis and a low Cronbach’s alpha (α < 0.5) for internal consistency reliability. We found acceptable validity for this scale, with reasonable convergence validity where patients with worse hyperphagia symptoms missed more days from school, and caregivers indicated greater concerns on body esteem, family relationships, social life via the IWQOL-Kids (Parent Proxy). From the known-groups analysis, for patients with more severe hyperphagia symptoms, caregivers also indicated they adapted more weight management approaches and used more appetite suppressants. These findings suggest the SoH: Caregiver version may adequately capture the behavioral aspects of the hyperphagia through the lens of the caregiver as an observer-reported outcome measure. However, there exists room to refine and adjust the wording of the items and further evaluate potential redundancy across items to improve its reliability.

The findings from this study should be interpreted with caution. First, all information collected was caregiver-reported, and the reported diagnoses of BBS, obesity, and hyperphagia were not directly confirmed. As with any patient- or caregiver-reported data, the information may be subject to recall bias and subjective interpretation, potentially leading to under- or over-reporting of the severity or frequency of the hyperphagia-related symptoms and behaviors. Second, the cross-sectional nature of the study design prevented the assessment of test-retest reliability and establishment of responsiveness/ability to detect change and minimal clinically important difference thresholds of the questionnaires. Additional longitudinal research is needed to establish these measurement properties and to evaluate the tools’ effectiveness in detecting within-person change over time to inform treatment effectiveness from the patient and caregiver perspectives. Third, the study participants were recruited from the US, UK, Canada and Germany, with languages in English, French and German. There was no separate assessment specifically conducted for cultural and linguistic validation. Further research is warranted to enhance the generalizability of these tools. Fourth, although our exploratory assessment of the questionnaires showed acceptable reliability for the IoH subscales there is room for improvement for the SoH scale. To address this, the questionnaire could be further optimized by evaluating alternative recall periods and/or incorporating additional items to capture a broader range of hyperphagia symptoms and behaviors. However, all scales demonstrated satisfactory validity, albeit of cross-sectional data in the real-world setting. Additional research to further validate these measures for use in clinical trials is underway.

## Conclusion

Caregiver versions of the SoH and IoH demonstrated initial validity, reliability, and consistency for use in a real-world setting. Findings from this study provide preliminary evidence for the SoH/IoH as potentially suitable questionnaires to assess hyperphagia signs/symptoms and impacts in patients with BBS or alike conditions and their caregivers. Additional studies to further improve and validate psychometric properties of the questionnaires, including the SoH and IoH Patient versions across other health care settings and over repeated longitudinal measures, are needed.

## Data Availability

Anonymized patient data and study documents can be requested from Rhythm Pharmaceuticals via the corresponding author for further research.

## Abbreviations

BBS: Bardet-Biedl syndrome
BMI: Body mass index
EFA: Exploratory factor analysis
MC4R: Melanocortin-4 receptor
IoH: Impacts of Hyperphagia
IWQOL: Impact of Weight on Quality of Life
KMO: Kaiser-Meyer-Olkin
PRO: Patient-reported outcome
PROMIS: Patient-Reported Outcomes Measurement Information System
RIOFS: Revised Impact on Family Scale
SD: Standard Deviation
SoH: Symptoms of Hyperphagia
WPAI: Work Productivity and Activity Impairment
